# The public’s antibiotic use behavioural patterns and their determinants for upper respiratory tract infections: a latent class analysis based on consumer behaviour model in China

**DOI:** 10.3389/fpubh.2023.1231370

**Published:** 2023-12-15

**Authors:** Rujiao Lin, Lixia Duan, Chaojie Liu, Dan Wang, Xinping Zhang, Xi Wang, Xinyi Zhang, Qianning Wang, Shuangjiang Zheng, Chenxi Liu

**Affiliations:** ^1^School of Medicine and Health Management, Tongji Medical School, Huazhong University of Science and Technology, Wuhan, Hubei, China; ^2^School of Psychology and Public Health, La Trobe University, Melbourne, VIC, Australia; ^3^School of Management, Hubei University of Chinese Medicine, Wuhan, Hubei, China; ^4^Department of Medical Affairs, The First Affiliated Hospital of Chongqing Medical University, Chongqing, China

**Keywords:** antibiotic, capacity-opportunity-motivation behaviour, consumer behaviour model, upper respiratory tract infections, the public

## Abstract

**Background:**

The irrational use of antibiotics among the public is a major contributor to antimicrobial resistance (AMR), which is a serious global threat. Prior studies have demonstrated that there are different behavioural patterns regarding antibiotic use among the public, and targeted interventions for subgroups with different behavioural patterns may be more effective. Thus, this study aimed to identify the public’s behavioural patterns of antibiotic use for upper respiratory tract infections (URTIs) and their influencing factors.

**Methods:**

A cross-sectional survey was conducted among the general population in Chongqing, China. Consumer decision-making (Consumer Behaviour Model, CBM) was used to assess the public’s behaviours regarding antibiotic use, including need recognition, information searching, alternative evaluation, obtaining antibiotics, antibiotic consumption, and postuse evaluation. Furthermore, a latent class analysis was used to identify the underlying behavioural patterns among the public. The identified behavioural patterns of antibiotic use were further linked with individuals’ capacity, opportunity, and motivation factors of antibiotic use based on a multinominal logistic regression to explore possible determinants.

**Results:**

A total of 815 respondents were enrolled in the study. The public’s irrational use of antibiotics was prevalent, including antibiotic self-medication (39.63%), nonprescription antibiotic purchasing (59.02%), and early stopping of antibiotic prescriptions (76.56%). Participants had inadequate knowledge of antibiotics (Mean = 2.33, SD = 1.71), reported high availability to antibiotics (Mean = 7.13, SD = 2.41), held strong belief in antibiotic effectiveness (Mean = 10.29, SD = 2.71), and demonstrated a high perceived threat of AMR (Mean = 12.30, SD = 3.20). Four behavioural patterns regarding antibiotic use for URTIs were identified, namely, “antibiotic self-medicators” (*n* = 165, 20.25%), “formal health care seekers” (*n* = 216, 26.50%), “various treatment users” (*n* = 198, 24.20%), and “self-medication without antibiotics” (*n* = 236, 28.96%). Individuals’ self-efficacy of antibiotic use, belief in antibiotic effectiveness, awareness of antibiotic side effects, perceived antibiotic availability, social influence, and demographics (age, education, medical insurance, and having a medical background) were significantly associated with the public’s different behavioural patterns of antibiotic use for URTIs.

**Conclusion:**

This study calls for collaborative efforts among the public, physicians, policy makers, and the implementation of precise and multifaceted interventions to effectively reduce irrational use of antibiotics in the public. Such interventions include identifying subgroups within the public to provide more targeted education about antibiotics and the management of URTIs, reinforcing the regulation of antibiotic dispensing, and improving physicians’ rational antibiotic prescriptions.

## 1 Introduction

Antimicrobial resistance (AMR) is considered one of the biggest threats to public health and economic development worldwide (1, 2). It is estimated that AMR was associated with 4.95 million deaths, including 1.27 million directly attributable deaths in 2019 (1). Without effective countermeasures, AMR is projected to become the leading cause of death, resulting in 10 million deaths every year with a total of 2.5–3% loss of global gross domestic product (GDP) by 2050 (3).

To address this issue, it is crucial to promote the rational use of antibiotics among the public (4, 5). Existing studies have shown that individuals’ antibiotic use not only significantly accelerates AMR (6, 7) but also induces more inappropriate antibiotic prescriptions among physicians (8–11). However, the irrational use of antibiotics among the public is prevalent worldwide (12–14), especially for treating upper respiratory tract infections (URTIs) and in developing countries. For example, it has been estimated that 50% of antibiotics were purchased without prescriptions in most parts of the world (15), and that non-prescription antibiotic use accounted for 19–100% of overall antibiotic use outside of northern Europe and North America (14). Moreover, nonadherence to prescribed antibiotic therapy is also prevalent (12, 13, 16), which further increases the risk of AMR (17).

To inform effective interventions, it is important to understand why people irrationally use antibiotics (18). Existing studies have shown that antibiotic use behaviours among the public include a variety of behaviours (18), such as information seeking, assessing alternatives, and antibiotics acquisition. Furthermore, antibiotic use behaviours among the public are highly diversified, and the combination of these behaviours leads to several relatively fixed patterns of behaviours when people use antibiotics, namely “antibiotic use behavioural patterns” (19, 20). For example, while some individuals prefer to obtain antibiotics through physician’s’ prescriptions, others may obtain and utilize antibiotics without seeking medical advice (19, 21).

Despite the variations in antibiotic use behaviours among the public, existing studies often provided partial depictions of people’s antibiotic use behaviours and neglected the heterogeneity within the public (13, 22–24). On the one hand, existing researches mainly focused on examining the impact of knowledge and attitude on irrational antibiotic use through knowledge, attitude, and practices (KAP) model (22). However, enhancing knowledge and attitude alone did not necessarily result in a reduction of irrational antibiotic use (25, 26). On the other hand, several studies have explored people’s different behavioural patterns of antibiotic use based on qualitative design (20, 27) or by analysing several antibiotic use behaviours (19, 21, 28, 29). This resulted in mixed findings due to the differences in research outcomes, settings, and populations (30). Furthermore, these studies have often failed to establish connections with potential influencing factors, the underlying factors that can impact the individuals to form a specific antibiotic use behavioural pattern. Some studies have tried to link behavioural patterns with some influencing factors, such as perceived severity of having an antibiotic resistant infection (19, 21) and the perceived efficacy of antibiotics to treat non-bacterial illness (19, 21, 28). However, this still leaves the underlying causes of the diverse antibiotic use behaviours unclear.

China is one of the largest producers and users of antibiotics worldwide (31, 32). The public’s irrational use of antibiotics has increasingly become the main contributor to antibiotic abuse and significantly boosted AMR in China (33), especially in the context that physicians’ irrational prescriptions of antibiotics have significantly decreased in recent years (34, 35). Therefore, the current study aimed to answer the following research questions:

RQ1: What are the underlying antibiotic use behavioural patterns among Chinese residents?

RQ2: What are the potential determinants that influence individuals’ antibiotic use behavioural patterns?

## 2 Participants and methods

### 2.1 Theoretical framework

**Figure 1:**
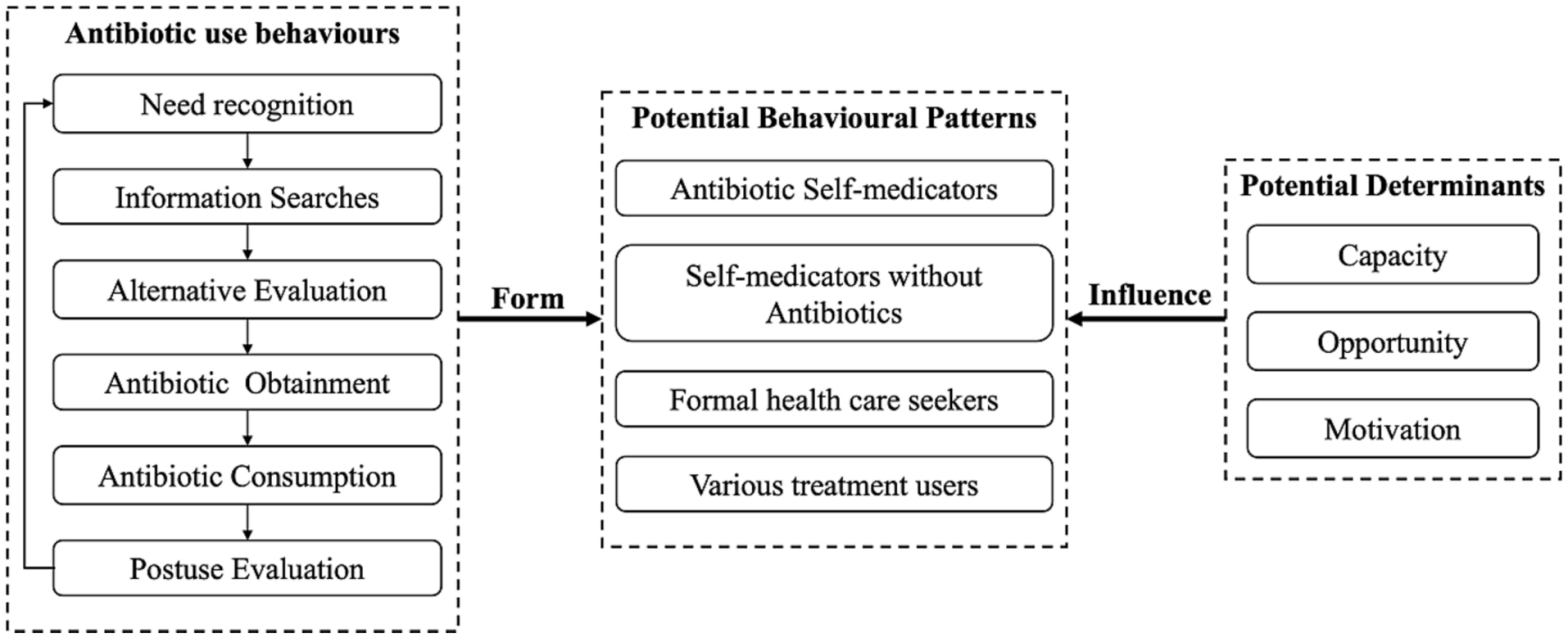
Theoretical framework.

This study was based on the Consumer Behaviour Model (CBM) (36) and Capacity-Opportunity-Motivation Behaviour (COM-B) framework, which have been used to guide existing evidence synthesis regarding the public’s antibiotic use behaviours and their potential determinants (for details, please see publication elsewhere (18)). Briefly, to address RQ1, the CBM was used to depict the whole process of the public’s behaviours regarding antibiotic use and served as indicators to identify the public’s behavioural patterns through latent class analysis (LCA). By using LCA, subgroups were identified based on shared patterns of antibiotic use behaviours (37). To address RQ2, the COM-B was applied to identify potential influencing factors, which was further linked with the behavioural patterns through multinominal logistic regression (Figure 1).

Based on the CBM, people’s antibiotic use behaviours involve six stages, namely, need recognition, information searches, alternative evaluation, antibiotic obtainment, antibiotic consumption, and postuse evaluation (18). Specifically, people first assess the severity of their diseases (need recognition) and acquire information regarding coping strategies from various sources (information searches). They assess potential coping strategies (alternative evaluation) and obtain antibiotics from various channels if necessary (antibiotic obtainment). During consumption, adjustment of usage may be applied (antibiotic consumption), and then people generate postuse evaluations to inform future use of antibiotics (postuse evaluation).

On the other hand, based on the COM-B, people’s capacity (capacity to engage in antibiotic use, involving an individual’s skills and knowledge), opportunity (external drivers to enable or to prompt antibiotic use, such as antibiotic availability) and motivation (intrinsic driving forces that induce antibiotic use, such as personal beliefs) factors are potential determinants of their different antibiotic use behavioural patterns (18, 38).

### 2.2 Setting

This study was conducted in Chongqing, one of the four municipalities (provincial-level divisions) in China. Chongqing is one of the inland cities in western China, with a population of 32.12 million (2.28% of all populations in China) (39) and upper-middle socioeconomic development (*per capita* GDP: $13,490 in 2022) based on the World Bank Classification, ranking in the middle of all province-level areas (13/34) in China (40).

Regarding the prevalence of the general population’s irrational antibiotic use in Chongqing, a meta-analysis revealed that people from western China commonly demand antibiotic prescriptions (65%), purchase antibiotics without prescriptions (70%), and have prophylactic use of antibiotics (47%) (13). This situation is also applied to parental antibiotic use for their children, including antibiotic self-medication (43.2%) and nonprescription antibiotic purchasing (70.4%) (41).

### 2.3 Sample size and sampling

The sample size was calculated based on the following formula (42):


$$n=\frac{{Z_{\alpha /2}}^{2}\times p\times \left(1-p\right)}{\delta^{2}}\times \left[1+\left(M-1\right)\times ICC\right]$$

According to previous studies, the prevalence of the public’s irrational antibiotic use behaviours ranged from 36.5% to 81.8% (40). Considering α (significance level) = 0.05, δ (tolerance) = 0.05, M (number of participants in each cluster) = 35, and intraclass correlation coefficient (ICC) = 0.02, the sample size was required to be larger than 646. We estimated that the invalid response rate would range from 10 to 12%, so the final sample size was calculated as 735, with 21 clusters.

A two-stage cluster random sampling strategy was used in the current study. In the first stage, Yuzhong District, Tongnan District, and Chengkou District were selected randomly, respectively representing high, middle, and low socioeconomic levels in Chongqing (39). In the second stage, seven to nine primary care institutions (urban community health centres and rural township health centres) were randomly selected from all primary care institutions in each area. This resulted in a sample of 23 institutions.

The inclusion and exclusion criteria of participants were as follows: (1) more than 18 years old; (2) having experienced symptoms of URTIs in the past 6 months, for example, cough, sneezing, and fever; (3) no mental disorders or other serious illnesses; and (4) consent to participate in and able to complete the survey (with the help of data collectors if needed).

### 2.4 Measures

#### 2.4.1 Development of measures

A literature review and semistructured interviews were conducted to identify the antibiotic use behaviours and their influencing factors. First, studies exploring antibiotic use behaviours as well as their barriers, facilitators, or other factors were screened, included and synthesized through a systematic review (18). Eleven themes regarding antibiotic use behaviours were identified. Second, semistructured interviews were conducted with 15 participants in Wuhan city to further review the screened behaviours and factors for adaptability of quantitative surveys. The participants were asked about their past experiences and perceptions of using antibiotics for URTIs. A list of antibiotic use behaviours and factors was confirmed and refined. The final list of the chosen antibiotic use behaviours and factors is described below.

#### 2.4.2 Dependent variables

According to CBM (Figure 1), six stages of the public’s antibiotic use behaviours were measured, namely, need recognition, information searches, alternative evaluation, antibiotics obtainment, antibiotics consumption, and postuse evaluation. For each stage, two to four items were generated based on existing instruments (43) or drafted according to previous studies.

For need recognition, two items measured to what extent the public perceived URTIs as severe illnesses and were able to distinguish mild URTIs from severe ones. In terms of information searches, four items measured how often participants searched for information to cope with URTIs and which sources of information they used (health care providers, personal experience, family, friends, etc.). For alternative evaluation, four items measured the public’s common coping strategies for URTIs: home remedies for colds (such as staying hydrated and getting more rest), self-medication without antibiotics (such as 999 Cold Remedy Granules, an herbal medicine commonly used for treating colds in China), antibiotic self-medication, formal health care, and a combination of the above. Regarding antibiotics obtainment, three items assessed the sources of antibiotics (physicians’ prescription, nonprescription purchasing from retail pharmacies, family storage, etc.). Three items measured the public’s antibiotic consumption behaviours, specifically adjusting antibiotic uptakes, including overdosing (increasing the dosage due to concerns of poor effects), underdosing (decreasing the dosage due to concerns of side effects), and early stopping (stopping the course of antibiotics when the symptoms improved) of antibiotic prescriptions. Finally, four items measured postuse evaluation of antibiotics, including the effectiveness, side effects, relative effectiveness compared with other treatments, and future use of antibiotics for URTIs. Responses were based on 5-point Likert scales (for each item of dependent variables, see Supplementary material pages 1, 2, Table 1).

**Table 1:** Demographic characteristics of the respondents (*n* = 815).

Characteristics	*N*	%
Age (years)		
>18 and <40	307	37.67%
≥40 and <65	357	43.80%
≥65	151	18.53%
Gender		
Male	319	39.14%
Female	496	60.86%
Education		
Primary school and less	275	33.74%
Junior school	188	23.07%
Senior school	114	13.99%
Bachelor’s degree and more	238	29.20%
Annual household income (Chinese Yuan)		
[0,20,000)	224	27.48%
[20,000,40,000)	159	19.51%
[40,000,60,000)	119	14.60%
[60,000,80,000)	65	7.98%
[80,000,100,000)	73	8.96%
[100,000,120,000)	62	7.61%
[120,000,140,000)	25	3.07%
[140,000,160,000)	11	1.35%
≥160,000	77	9.45%
Medical insurance		
New Cooperative Medical Scheme (NCMS)	342	41.96%
Urban Employee Basic Medical Insurance (UEBMI)	228	27.98%
Medical Assistance for Urban Residents (MAUR)	226	27.73%
Others	19	2.33%
Self-rated health		
Excellent	286	35.09%
Good	249	30.55%
Moderate	199	24.42%
Poor	81	9.94%
**Have medical background**	144	17.67%
**Have chronic diseases**	404	49.57%

#### 2.4.3 Independent variables

##### 2.4.3.1 Capacity

Knowledge and self-efficacy regarding antibiotic use were used to assess the public’s capacity for rational use of antibiotics. Eight true-false items assessed whether the public knew indicators of antibiotic use, causes of AMR, and common misconceptions about antibiotics and AMR. Five items measured on a 5-point Likert scale indicated the public’s self-efficacy for the rational use of antibiotics, for example, “I’m confident that I have sufficient knowledge of the rational use of antibiotics” (44–46).

##### 2.4.3.2 Opportunity

Antibiotic availability and social influence were used to assess the public’s opportunity to use antibiotics rationally. Antibiotic availability was measured by three items asking participants how easily they can obtain antibiotics from retail pharmacies (without prescriptions), from family members, and from friends (47, 48). In terms of social influence, two items assessed to what extent the public’s antibiotic use was influenced by physicians, pharmacists, families, and social norms (41). The responses were measured on 5-point Likert scales.

##### 2.4.3.3 Motivation

The expected positive and negative effects of antibiotics, perceived threat of URTIs and AMR, and rationale of treatment choice were measured on 5-point Likert scales to evaluate the public’s motivation to use antibiotics rationally. Four and three items measured the positive (for example, shortening URTI duration) and negative effects (for example, side effects) of antibiotics, respectively. Perceived threats of URTIs and AMR were measured by three and five items, respectively (49, 50), by asking participants to what extent they thought URTIs and AMR may threaten themselves, their families, or public health. For the rationale of treatment choice, participants were asked to what extent they agree to have antibiotic use for common colds just in case, for example, “although I am not entirely certain of antibiotics’ effectiveness, I would use them to treat a common cold as a precautionary measure” (For details on the independent variables, please see Supplementary material pages 3, 6, Table 2).

**Table 2:** Respondents’ behaviours regarding antibiotic use based on CBM.

Behaviours	Yes (*N*%)	No (*N*%)
Need recognition		
Perceive URTIs as severe illnesses	187 (22.94%)	628 (77.06%)
Able to distinguish severe URTIs from mild ones	439 (53.87%)	376 (46.13%)
Information searching		
Search information for treatment of URTIs	148 (18.16%)	667 (81.84%)
Use personal experience as information source	531 (65.15%)	284 (34.85%)
Use physicians as information source	711 (87.24%)	104 (12.76%)
Use others (family, friends, social media, etc.) as information source	268 (32.88%)	547 (67.12%)
Alternative evaluation		
Use home remedies for colds to cope with URTIs	416 (51.04%)	399 (48.96%)
Use self-medication without antibiotics to cope with URTIs	464 (56.93%)	351 (43.07%)
Use antibiotic self-medication to cope with URTIs	323 (39.63%)	492 (60.37%)
Use formal health care seeking to cope with URTIs	361 (44.29%)	454 (55.71%)
Antibiotics obtaining		
Obtain antibiotics from physicians’ prescriptions	675 (82.82%)	140 (17.18%)
Obtain antibiotics from retail pharmacies without prescriptions	481 (59.02%)	334 (40.98%)
Obtain antibiotics from other ways (storage, family, friends, etc.)	298 (36.56%)	517 (63.44%)
Antibiotics consuming		
Have increased dosing of antibiotics	108 (13.25%)	707 (86.75%)
Have decreased dosing of antibiotics	165 (20.25%)	650 (79.75%)
Early stopping of antibiotic prescriptions	624 (76.56%)	191 (23.44%)
Post-consumption evaluating		
Perceive antibiotics as effective for URTIs	456 (55.95%)	359 (44.05%)
Perceive severe side effects of antibiotics for URTIs	141 (17.30%)	674 (82.70%)
Perceive antibiotics as better one compared with other medicines for URTIs	417 (51.17%)	398 (48.83%)
Expect to use antibiotic for future URTIs	202 (24.79%)	613 (75.21%)

#### 2.4.4 Demographic characteristics

Respondents’ demographic characteristics were also collected, including gender, age, education, occupation, annual household income, medical insurance, chronic diseases, self-rated health status, and medical background (please see Supplementary material page 7, Table 3).

**Table 3:** Influencing factors regarding antibiotic use based on COM-B.

Factors (overall scores)	Mean ± SD
Capacity	
Knowledge (0–8)	2.33 ± 1.71
Self-efficacy (0–20)	9.84 ± 3.74
Opportunity	
Antibiotic availability (0–12)	7.13 ± 2.41
Social influence (0–8)	4.09 ± 2.00
Motivation	
Expected positive effects of antibiotics (0–16)	10.29 ± 2.71
Expected negative effects of antibiotics (0–12)	6.47 ± 2.49
Rationale of treatment choice (0–16)	5.59 ± 2.69
Perceived threat of URTIs (0–12)	6.40 ± 3.11
Perceived threat of AMR (0–20)	12.30 ± 3.20

#### 2.4.5 Pilot study

The development of the survey instrument followed existing guidelines (51). A pilot study was conducted in Wuhan among seventeen respondents with different demographic characteristics (gender, age, and income) to check the readability as well as instrument validity and reliability. Some items were revised, added, or removed based on the results and feedbacks from the pilot study. Factor analysis and Cronbach’s alpha were used to check validity and reliability. The pilot study confirmed the face, content validity, and reliability (Cronbach’s alpha: 0.60–0.89, please see Supplementary materials page 8, Table 4).

**Table 4:** Results of multinomial logistic regression on factors associated with behavioural patterns of antibiotic use.

Indicators	Model 1, RRR (95%CI)			Model 2, RRR (95%CI)		
	Various treatment users	Antibiotic self-medicators	Formal health care seekers	Various treatment users	Antibiotic self-medicators	Formal health care seekers
Capacity						
Knowledge	1.103 (0.971, 1.254)	0.854^*^ (0.732, 0.995)	0.807^**^ (0.696, 0.935)	0.992 (0.860, 1.144)	0.918 (0.769, 1.096)	0.947 (0.800, 1.121)
Self-efficacy	1.016 (0.958, 1.077)	1.126^**^ (1.052, 1.205)	0.942 (0.885, 1.003)	1.041 (0.975, 1.112)	1.114^**^ (1.037, 1.198)	0.928^*^ (0.866, 0.994)
Opportunity						
Antibiotic availability	1.009 (0.920, 1.106)	1.212^***^ (1.088, 1.350)	0.949 (0.860, 1.046)	1.021 (0.924, 1.128)	1.166^**^ (1.042, 1.304)	0.910 (0.818, 1.012)
Social influence	1.329^***^ (1.176, 1.501)	1.030 (0.910, 1.166)	0.921 (0.820, 1.035)	1.203^**^ (1.050, 1.379)	1.141 (0.997, 1.307)	1.029 (0.907, 1.168)
Motivation						
Expected positive effects of antibiotics	1.083 (0.999, 1.174)	1.249^***^ (1.132, 1.378)	1.246^***^ (1.138, 1.364)	1.076 (0.986, 1.175)	1.216^***^ (1.098, 1.346)	1.183^***^ (1.076, 1.301)
Expected negative effects of antibiotics	1.104 (0.991, 1.231)	0.797^***^ (0.711, 0.893)	0.736^***^ (0.660, 0.821)	1.123 (0.995, 1.268)	0.822^***^ (0.728, 0.928)	0.786^***^ (0.701, 0.881)
Rationale of treatment choice	1.083 (0.990, 1.184)	1.041 (0.947, 1.145)	1.058 (0.966, 1.158)	1.138^*^ (1.027, 1.260)	1.048 (0.949, 1.158)	1.051 (0.956, 1.156)
Perceived threat of URTIs	1.040 (0.968, 1.117)	1.086^*^ (1.006, 1.172)	1.174^***^ (1.093, 1.261)	1.087^*^ (1.004, 1.178)	1.067 (0.983, 1.158)	1.146^***^ (1.062, 1.237)
Perceived threat of AMR	0.968 (0.897, 1.045)	0.951 (0.876, 1.033)	1.002 (0.924, 1.085)	0.990 (0.913, 1.075)	0.943 (0.863, 1.030)	1.012 (0.930, 1.102)

*N* = 815. The reference group was class 3 (“self-medicators without antibiotics”). Model 1 was a coarse estimation, and Model 2 was adjusted for demographic characteristics. RRR = relative risk ratios. CI = confidential interval.

* *p* < 0.05,

** *p* < 0.01,

*** *p* < 0.001.

### 2.5 Data collection

A cross-sectional survey was conducted from July 24 to August 4, 2022. A self-administered paper-based questionnaire was used to collect the data.

To recruit eligible participants, two data collectors were sent to each primary care facility. Patients and their companions were approached and were invited to participate in the study if they met the inclusion criteria. Each eligible participant was informed about the background and aim of the survey, ways of filling out the questionnaire, and the principle of confidentiality.

For quality control, returned questionnaires were immediately checked on the spot by data collectors, and missing items were filled in. Questionnaires were considered ineligible if responses contained unanswered items, all choices were the same, or they were returned in a very short time (less than two minutes). On average, the survey took 20 min, and respondents received a token gift (roughly $1.65) upon completion of the survey. Finally, a total of 955 questionnaires were distributed, and 906 were returned. A total of 815 responses were regarded as eligible for further analysis (response rate: 85.34%).

### 2.6 Statistical analysis

For items measuring antibiotic use behaviours, responses were coded as dichotomous variables (strongly agree/agree = 1 and other responses = 0). For measurements of the public’s capacity, opportunity, and motivation factors for antibiotic use (despite the knowledge as the sum of respondents’ correct answers), responses were coded from 0 to 4, with higher scores indicating higher levels of measured constructs; for example, higher scores indicated a higher expected positive/negative effect of antibiotic use.

LCA was performed to identify the underlying behavioural patterns of antibiotic use among respondents based on maximum likelihood estimation. We successively hypothesized that there were one to seven potential behavioural patterns of antibiotic use among respondents and compared the model fit indices to identify the best fit model. The model fit indices included the Akaike information criterion (AIC), Bayesian information criterion (BIC), adjusted Bayesian information criterion (aBIC), likelihood ratio (LR) test, entropy, and minimum class proportion (52). Furthermore, each respondent was classified into one exclusive antibiotic use behavioural pattern according to the best fit model. Finally, the identified antibiotic use behavioural pattern of each respondent was used as the dependent variable and further linked with one’s capacity, opportunity, and motivation factors regarding antibiotic use based on a multinominal logistic regression. Demographic characteristics were further adjusted to ensure the robustness of their effects.

Data analysis was conducted based on STATA (Version 16.0) and Mplus (Version 8). A *p* value of less than 0.05 was considered to be statistically significant.

## 3 Results

### 3.1 Characteristics of respondents

Nearly half of the respondents were aged between 40 and 65 years old (43.8%), and most were female (60.86%). Over half of the respondents had finished primary or secondary school (56.81%). The annual household income of two-thirds of respondents (61.59%) was below￥60,000 (roughly $8,736). Almost all respondents had basic medical insurance (97.67%), and respondents commonly rated their health status as excellent (35.09%) or good (30.55%). Among all the respondents, 17.67% reported having a medical background, and 49.57% reported having chronic diseases.

### 3.2 Behaviours regarding antibiotic use

According to the six stages of behaviours regarding antibiotic use based on CBM, most respondents (*n* = 628, 77.06%) did not consider URTIs as severe illnesses, and more than half of them were able to differentiate mild URTIs from severe ones (*n* = 439, 53.87%). Approximately one-fifth of respondents indicated that they often/always searched for information about the treatments of URTIs (*n* = 148, 18.14%). Physicians (*n* = 711, 87.24%) and personal experience (*n* = 531, 65.15%) were the two main information sources. To cope with URTIs, self-medication without antibiotics was most commonly used among respondents (*n* = 464, 56.93%), followed by home remedies for colds (*n* = 416, 51.04%), formal health care seeking (*n* = 361, 44.29%) and self-medication with antibiotics (*n* = 323, 39.63%). Antibiotics were commonly obtained with physicians’ prescriptions (*n* = 675, 82.82%) and retail pharmacies without prescriptions (*n* = 481, 59.02%). Respondents commonly showed early stopping of antibiotic prescriptions (*n* = 624, 76.56%). In terms of postuse evaluation, over half of the respondents reported that antibiotics were better than other medicines (*n* = 415, 51.57%) and effective for URTIs (*n* = 456, 55.95%). Approximately one-fourth of respondents often/always expected to use antibiotics for URTIs in the future (*n* = 202, 24.79%).

### 3.3 Influencing factors regarding antibiotic use

Based on COM-B, the public had inadequate knowledge of antibiotics, with less than three correct answers out of eight items (Mean = 2.33, Standard Deviation/SD = 1.71). However, they tended to have confidence in their ability to use antibiotics rationally (self-efficacy, mean = 9.84, SD = 3.74).

For opportunity factors, the respondents perceived a moderately high level of antibiotic availability (Mean = 7.13, SD = 2.41), which means that they could easily obtain antibiotics from retail pharmacies (without prescriptions), family, and friends. The respondents also perceived strong social influence to use antibiotics irrationally (Mean = 4.09, SD = 2.00), and almost half of them had received recommendation to use antibiotics from their family and friends (*n* = 346, 42.46%) or retail pharmacies (n = 396, 48.59%).

Finally, for motivation factors, the respondents tended to have positive expectations of the effectiveness (mean = 10.29, SD = 2.71) and moderate awareness of the side effects (mean = 6.47, SD = 2.49) of antibiotics. The respondents tended to not use antibiotics as a precautionary measure (Mean = 5.59, SD = 2.69). However, more than one-third of them still used antibiotics without indications of URTIs (overall score > 6, *n* = 304, 37.30%). The public generally perceived the threats of URTIs (mean = 6.40, SD = 3.11) and AMR (mean = 12.30, SD = 3.20).

### 3.4 Behavioural patterns of antibiotic use

Based on the LCA, a four-class model was selected based on model fit indices (AIC = 17716.57, BIC = 18106.93, aBIC = 17843.36, Entropy = 0.756, LR = 0.003, Minimum class proportion = 20.62%). In addition, high classifications of behavioural patterns among different individuals were confirmed in the four-class model based on posterior probabilities (>0.800; see Supplementary material Tables 5, 6).

Based on the results of LCA, respondents were divided into four behavioural patterns of antibiotic use, namely, “antibiotic self-medicators” (*n* = 165, 20.25%, referred to those who significantly preferred to use and purchased antibiotics without prescriptions), “self-medicators without antibiotics” (*n* = 236, 28.96%, referred to those who preferred to use non-antibiotic treatments for URTIs), “formal health care seekers” (*n* = 216, 26.50%, referred to those who preferred formal health care for URTIs) and “various treatment users” (*n* = 198, 24.20%, referred to those who preferred various treatments with and without antibiotics for URTIs).

Among them, “antibiotic self-medicators” rarely searched for information about URTI treatment (conditional probability, CP: 0.140) and mainly referred to their own experience (CP: 0.791). They preferred antibiotic self-medication to treat URTIs (CP: 0.842) and commonly obtained antibiotics from retail pharmacies without prescriptions (CP: 0.848). They also had the highest possibility of using antibiotics in the future (CP: 0.520), believing that antibiotics had positive effects on URTIs (CP: 0.791) and few side effects (CP: 0.033).

However, “self-medicators without antibiotics” commonly perceived URTIs as less severe diseases (CP: 0.078) and mainly chose home remedies for colds (CP: 0.688) and self-medication without antibiotics (CP: 0.789). They showed the lowest likelihood of self-medication with antibiotics (CP: 0.167) and had the lowest expectation to use antibiotics for URTIs in the future (CP: 0.025).

“Formal health care seekers” perceived the highest severity of URTIs (CP: 0.411) and commonly used formal health care as a coping strategy for URTIs (CP: 0.802). Physicians were their main source for information (CP: 1.000) and antibiotics (CP: 0.989). They rarely increased (CP: 0.090) or reduced (CP: 0.111) the dosages of antibiotics but normally showed early stopping of antibiotic prescriptions (CP: 0.824). Like “antibiotic self-medicators,” “formal health care seekers” also expected to use antibiotics in the future (CP: 0.382), believing that such medicine had positive effects on URTIs (CP: 0.770) and few side effects (CP: 0.086).

**Figure 2:**
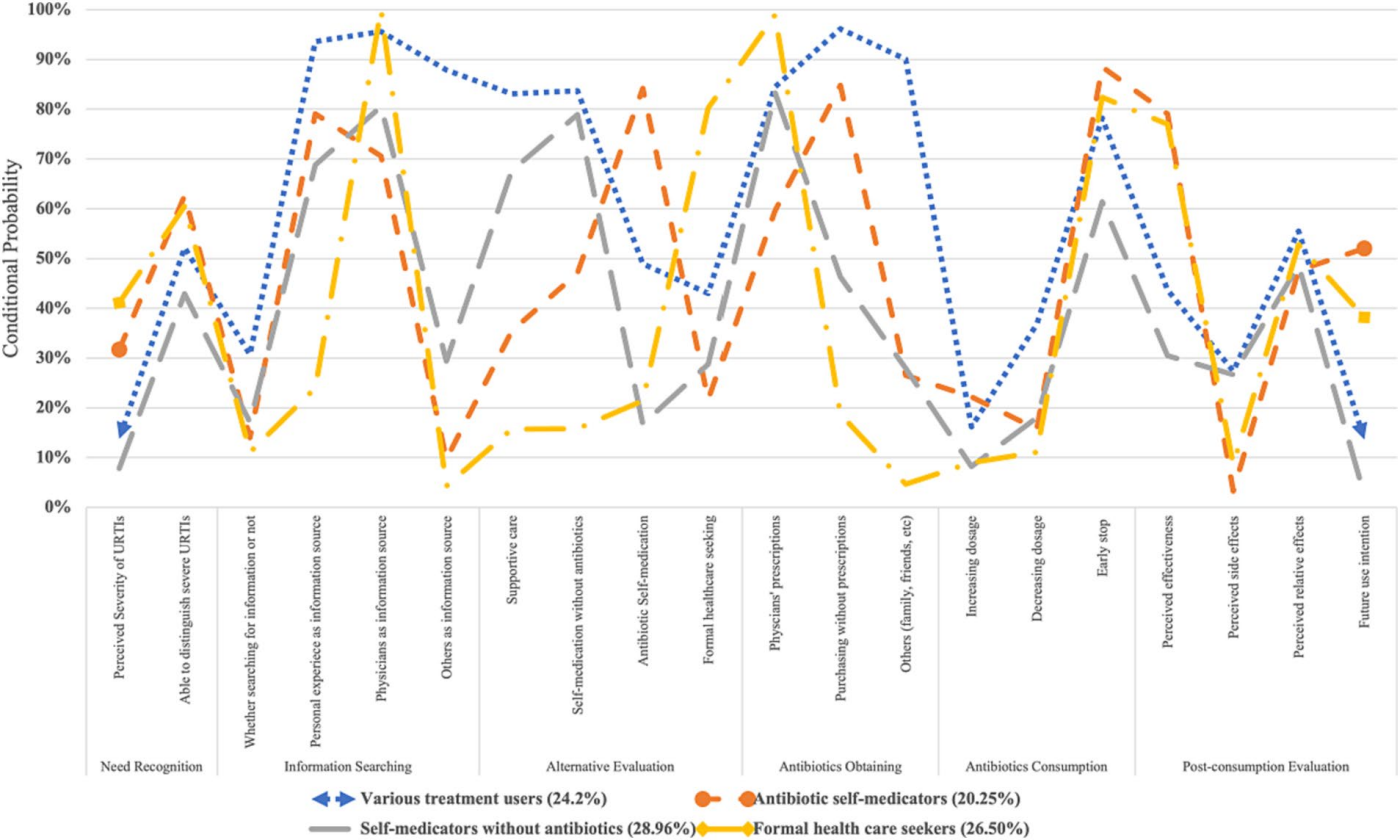
LCA results for behavioural patterns of antibiotic use for URTIs among the public.

Finally, “various treatment users” referred to various sources of information, including physicians (CP: 0.956), personal experience (CP: 0.936), and social networks, relatives and friends (CP: 0.879). They preferred to use multiple treatments for URTIs, including home remedies for colds (CP: 0.831), self-medication without antibiotics (CP: 0.837), sometimes self-medication with antibiotics (CP: 0.489), and formal health care (CP: 0.430; Figure 2).

### 3.5 Multinomial logistic regression

A multinomial logistic regression model (Table 4) was used to examine the relationships between respondents’ behavioural patterns and their capacity, opportunity, and motivation factors for antibiotic use without (Model 1) or with (Model 2) the adjustment of demographic characteristics, in which “self-medicators without antibiotics” class was treated as the reference group.

Compared with “self-medicators without antibiotics,” “antibiotic self-medicators” were more common among respondents with lower education (relative risk ratios, RRR = 0.600, *p* = 0.006) and with a basic medical insurance system for urban employees (RRR > 1, *p* < 0.01). This behavioural pattern was associated with higher self-efficacy of antibiotic use (RRR = 1.114, *p* = 0.003), more positive belief in the effectiveness of antibiotics (RRR = 1.216, *p* < 0.001), lower awareness of side effects of antibiotics (RRR = 0.822, *p* = 0.001), and a higher level of antibiotic availability (RRR = 1.166, *p* = 0.007).

In contrast, “formal health care seekers” were more likely to be identified among respondents with lower education (RRR = 0.485, *p* < 0.001). They had a higher perceived threat of URTIs (RRR = 1.146, *p* < 0.001), higher self-efficacy of antibiotic use (RRR = 0.928, *p* = 0.034), more positive beliefs in antibiotic effectiveness (RRR = 1.183, *p* = 0.001) and lower awareness of the side effects of antibiotics (RRR = 0.786, *p* < 0.001).

Finally, “various treatment users” were more likely to be observed among younger respondents (RRR = 0.965, *p* = 0.003) and those without a medical background (RRR = 0.447, *p* = 0.007). Higher social influence (RRR = 1.203, *p* = 0.008), higher perceived threat of URTIs (RRR = 1.138, *p* = 0.013), and more antibiotic usage as a precautionary measure (RRR = 1.087, *p* = 0.041) significantly increased the likelihood of respondents to show the “various treatment users” behavioural pattern.

## 4 Discussion

### 4.1 Main findings

Based on the CBM, this study comprehensively depicted the six stages of the public’s behaviours regarding antibiotic use, including need recognition, information searches, alternatives assessment, antibiotic obtainment, antibiotic consumption, and postuse evaluation. The results of the current study showed that the irrational use of antibiotics is prevalent in China. Approximately 40% of the respondents reported antibiotic self-medication for URTIs, and 59.02% had obtained antibiotics from retail pharmacies without prescriptions. Overdosing (13.25%), underdosing (20.25%) and early stopping (76.56%) of antibiotic prescriptions were also prevalent. An LCA model was further applied, and four behavioural patterns regarding the public’s antibiotic use were identified, including “antibiotic self-medicators,” “formal health care seekers,” “various treatment users” and “self-medicators without antibiotics.” Individuals’ capacity, opportunity, and motivation factors for antibiotic use were further linked with individuals’ antibiotic use behavioural patterns. The results showed that individuals’ self-efficacy of antibiotic use, perceived threat of URTIs, beliefs in positive effects and side effects of antibiotics, and demographic characteristics (age, education, and medical background) significantly influenced behavioural patterns regarding antibiotic use.

### 4.2 Strengths and limitations

To the best of our knowledge, this is the first study, based on CBM, to systematically describe the public’s antibiotic use behaviours from the very beginning (need recognition) to the end (postuse evaluation). Based on LCA, the public’s underlying behavioural patterns regarding antibiotic use were further identified and linked with their capacity, opportunity, and motivation factors for antibiotic use, which deepens our understanding of how and why the public uses antibiotics to treat URTIs.

There are also some limitations. First, this was a cross-sectional study conducted in Chongqing, China, and focused solely on URTIs. Generalization to other populations or other diseases must be cautious. The study’s cross-sectional nature, conducted during the summer, may also introduce a seasonal bias by potentially overlooking variations in antibiotic use for URTIs across different seasons. Second, the results were based on respondents’ self-reported responses, which may be subject to response bias. Respondents may not accurately remember their previous experiences with antibiotic use or may omit details, given that the inclusion period was 6 months. They may also be inclined to provide socially acceptable answers, such as reporting a greater reliance on physicians’ prescriptions for antibiotic use. What’s more, respondents may have pre-existing misconceptions, regarding URTIs as inflammation and antibiotics as anti-inflammatory drugs. They may attribute the effectiveness and using experiences of anti-inflammatory drugs to antibiotics, potentially influencing the study’s results. Third, the use of Likert scales provided limited and predefined options, potentially simplifying the complex phenomena that determine and influence individuals’ antibiotic use behaviours.

To address these limitations, more evidence-based studies are necessary. For instance, the inclusion of cohort studies on actual behaviours could offer deeper insights into the actual behaviours of the public. Moreover, it is crucial to develop, implement, and rigorously evaluate interventions to reduce irrational antibiotic use among the public to effectively address the threats of AMR.

### 4.3 Interpretation

A high prevalence of irrational antibiotic use behaviours among the general public in this study was identified, with approximately 40% of the respondents admitting to self-medication with antibiotics. This result is consistent with previous studies conducted among rural residents in Anhui, China (46.3%) (30), and higher than those of residents in Wuhan, China (10.32%) (53) and the general population in China (23.9%) (54). The result also corresponds with the prevalence of antibiotic self-medication in low- and middle-income countries, such as Serbia (27.17%) (55) and Tanzania (58.0%) (56). More than half (59.02%) of the respondents obtained antibiotics from retail pharmacies without prescriptions, which is consistent with the results of a meta-analysis in China (47%) (13). This would be fuelled by the availability of online pharmacies, which contributed to greater over-the-counter (OTC) sales without prescriptions (57). The prevalence of nonadherence in this study, especially the early stopping of antibiotics prescriptions (76.56%), is higher than that reported in a meta-analysis, where the rate antibiotic nonadherence behaviours in China was 48% (13). This is also higher than the results reported in Jordan (32.10%) (58), France (35.5%) (59), Portugal (57.7%) (12), and Ethiopia (60.1%) (60). The irrational use of antibiotics by individuals identified in these studies can contribute to the spread of AMR and requires urgent and coordinated action.

Previous studies have demonstrated that there are different behavioural patterns regarding antibiotic use among the public (19–21, 23, 26). The behavioural patterns identified in the current study partially echoed with the results from previous segmentation of populations in America, in which the public was divided into antibiotic stockers (individuals who preferred to store and share antibiotic leftovers), demanders (individuals who preferred to request antibiotic prescriptions from health care providers), and stewards (individuals who preferred to show the least likelihood of having problematic antibiotic use behaviours) (19, 21, 25). “Antibiotic self-medicators” in the current studies shared the same behavioural patterns as stockers and demanders, while “self-medicators without antibiotics” were similar to stewards in the previous studies (19, 21, 25). However, “formal health care seekers” and “various treatment users” were missed in these studies due to limited studies on information searching and alternative assessment behaviours regarding antibiotic use. A systematic review found that interventions on changing knowledge, attitudes, or antimicrobial stewardship behaviours demonstrated a notable potential for schoolchildren and parents but not for the general public (61). This may be due to the high heterogeneity of the public, as shown in this study. This also calls for more precise designs of interventions to reduce irrational antibiotic use among the general public.

The reasons why individuals showed different behavioural patterns varied, which may partly explain the mixed results of influencing factors (knowledge, attitudes, etc.) on antibiotic use in existing studies (18).

The associations between the public’s motivation factors and antibiotic use were consistent with existing evidence that high levels of concerns regarding the severity of the condition and misbelief in the effectiveness of antibiotics increased the public’s irrational use of antibiotics. However, individuals’ capacity and opportunity factors showed mixed effects on one’s antibiotic use.

Our study did not find a significant association between antibiotic use and knowledge or perceived threat of AMR, which may be the mixed effects of knowledge and awareness in reducing irrational antibiotic use. Previous studies have shown that the public has an incomplete understanding of AMR (22) and antibiotic use (62). Although some studies found that improvements in knowledge promoted rational antibiotic use among the public in Belgium and France (63, 64), Roope et al. found that raising awareness of AMR was associated with an increased demand for antibiotics; this may be due to that such fear-appeal messages could trigger people to protect themselves (29, 65). A recent study in China also found that the awareness of AMR was high among social media users; however, inappropriate use and misconception of antibiotics remained commonly high (66).

As one of the most powerful predictors of health-related behaviours (67), self-efficacy of the rational use of antibiotics showed different effects on antibiotic self-medicators and formal health care seekers. People with higher self-efficacy are more confident about self-medication, while those with less self-efficacy tend to let physicians make medical decisions. However, the current study showed that, without adequate antibiotic use knowledge, such overconfidence indicated by the high level of self-efficacy would contribute to more irrational antibiotic behaviours, as shown in antibiotic self-medicators. Previous studies also demonstrated that people with higher self-efficacy are more confident in self-treating URTIs (68) and purchasing antibiotics from retail pharmacies (69) without prescriptions based on previous experience with diseases and medication.

In addition, social influence from physicians, retail pharmacies, friends, and family members had a positive effect on irrational antibiotic use. They are the main sources of health information about URTIs and antibiotic use (70). Common prescriptions from physicians and irrational antibiotic use within social networks (such as friends and family members) contribute to the social norms that support irrational antibiotic use (18).

AMR is a complex phenomenon heavily influenced by social determinants that lead to irrational antibiotic use. In this study, we investigated the impact of certain social factors (knowledge, antibiotic availability, and social influence) on antibiotic use. Although we did not find a significant effect of knowledge on antibiotic use, or consistent effects of antibiotic availability and social influence on antibiotic use, it’s noteworthy that existing studies have explored the roles of other social factors in shaping antibiotic use behaviours among the public across various contexts. Socio-economic and socio-cultural factors, for instance, have been identified as significant determinants influencing antibiotic consumption. Two systematic reviews have identified the impact of health system-related factors on self-medication with antibiotics among the public. These factors included barriers to health care, such as long waiting time for getting medical care, transportation issues, and limited financial resources for formal consultations (71, 72). Conversely, facilitators for antibiotic self-medication, such as affordability, easy accessibility of antibiotics from retail pharmacies, and the convenience associated with self-medication, have also been identified (71). Furthermore, various socio-cultural determinants have been found to influence antibiotic use, including the stigma of getting an infection when visiting hospitals (72), and patients’ work-related attitudes (e.g., the decision to continue working when afflicted by illnesses) (73). It is therefore imperative to integrate social factors in conjunction with evidence-based and theory-grounded framework to develop effective and appropriate interventions for promoting prudent antibiotic use.

### 4.4 Policy and practice implications

To reduce the irrational use of antibiotics, it is imperative for the public, physicians, and policy makers to collaborate jointly. And more accurate and multifaceted interventions are needed based on the results of the current study.

Because the public has several behavioural patterns regarding antibiotic use, it is vital to identify subgroups within the population to account for the heterogeneity of the public’s behaviours (74, 75) and to provide targeted education accordingly. Previous studies have explored the public’s antibiotic stewardship behavioural patterns in America and found it important to provide targeted interventions for subgroups with different behavioural patterns (19, 21, 24, 25). By identifying relevant subgroups, more accurate education and training programs can be developed targeting the determinants of the public’s different behavioural patterns of antibiotic use (76). For example, “antibiotic self-medicators” should be educated about the limited effect of antibiotics for URTIs, while “formal health care seekers” should be trained about the severity and causes of URTIs.

In addition, a general education program about safe antibiotic use and how to manage URTIs would also be important. Due to the self-limited nature of URTIs, previous studies have shown that people incorrectly attribute the recovery of URTIs to whatever they use, including antibiotics. The misconception of the effectiveness of antibiotics for URTIs, especially for “antibiotic self-medicators” and “various treatment users,” could induce unnecessary expectations of antibiotic use in the future. This situation could be more severe if there is abuse of antibiotics from physicians, which may further normalize the public’s irrational use of antibiotics (18).

Regulations and law enforcement need to be strengthened regarding retail dispensing, including prohibiting the sale of antibiotics without prescriptions and the regulation of antibiotic leftovers. Although the Chinese government has prohibited the sale of antibiotics without prescriptions (77), the present study revealed a prevalence of such behaviours among the public. Easy access to nonprescription antibiotics from retail pharmacies has been commonly observed in most areas of China (30). Previous studies have also reported nonprescription sales of antibiotics in low- and middle-income countries, despite legislation against such practices (78–80). These practices are driven by various reasons, including pressure from patients and financial motives. Community pharmacists may fear losing clients who seek for antibiotics to competitors (81). Additionally, the low awareness of AMR among physicians and pharmacists also plays a role (82, 83). It appears that China needs to reinforce regulation of the nonprescription sale of antibiotics, for example, regulating OTC antibiotic sales without prescriptions in both offline and online pharmacies, increasing training for physicians and pharmacists about AMR, and providing guidelines for appropriate antibiotic dispensing. However, existing studies showed that such interventions showed mixed effects (84), and whether they would be effective in China warrants future study. Moreover, it is also crucial to regulate antibiotic leftovers, as higher levels of self-medication were associated with having leftover antibiotics (85–87). Effective measurements include dispensing antibiotics in an exact number of tablets according to the prescriptions, and collecting or encouraging the return of unused or expired antibiotics.

Finally, health care providers also play a role in changing the public’s irrational antibiotic use behaviours, as they are commonly regarded as information sources for health. On the one hand, it is important for patients to reduce irrational expectations of antibiotics with the help of physicians and pharmacists. Physicians can manage patients’ expectations by using communication strategies to discourage irrational antibiotic use (88), especially for parents. Physicians also need to reduce irrational antibiotic prescriptions to help change the public’s misconception of the effectiveness of antibiotics for URTIs. Previous studies showed that nonevidence-based antibiotic prescriptions was frequent at the primary care level (88, 90). It is important to implement and reinforce the clinical guidelines for the rational use of antibiotics in clinical practice (91).

## 5 Conclusion

The irrational use of antibiotics among the public is prevalent in China, for which four different behavioural patterns were identified, namely, antibiotic self-medicators, formal health care seekers, various treatment users, and self-medicators without antibiotics. The antibiotic use behavioural patterns were further linked with individuals’ capacity, opportunity, and motivation factors. To reduce irrational antibiotic use among the public, it is imperative to design and implement more precise and comprehensive interventions that engage various stakeholders. It is essential to make campaign decisions that involve more relevant individuals effectively by identifying subgroups within the public that have different antibiotic use behaviours influenced by different factors. Additionally, given the intricate social context, it is crucial to involve stakeholders in efforts to reduce OTC antibiotic dispensing without prescriptions and enhance rational antibiotic prescribing practices among physicians. More studies and interventions involving both the supply side and demand side are needed to address the growing threats of AMR.

## Data availability statement

The original contributions presented in the study are included in the article/Supplementary material, further inquiries can be directed to the corresponding authors.

## Ethics statement

This study obtained ethics approval from the Research Ethics Committee of Tongji Medical College, Huazhong University of Science and Technology (No [2020] - S099). Informed consent was obtained from all subjects involved in the study.

## Author contributions

CheL, ChaL, and XinpZ conceptualized and designed the study. RL, LD, XW, QW, and XinyZ performed the data collection. CheL, DW, and RL performed formal data analysis. RL and CheL contributed to the writing of the manuscript. All authors contributed to the article and approved the submitted version.
