# Supplementary material for: The public’s antibiotic use behavioural patterns and their determinants for upper respiratory tract infections: a latent class analysis based on consumer behaviour model in China
Source: Front Public Health. 2023 Dec 15;11:1231370. doi: 10.3389/fpubh.2023.1231370 (PMC10754980; doi:10.3389/fpubh.2023.1231370)
Supplement: Supplementary file 1 [file Data_Sheet_1.docx]

**Table 1. Measures on antibiotic use based on CBM framework**

| **Need Recognizing** | **Strongly**  **Agree** | **Agree** | **Neither** | **Disagree** | **Strongly**  **Disagree** |
| --- | --- | --- | --- | --- | --- |
| Do you think that common cold is a kind of severe illness? |  |  |  |  |  |
| Can you recognize when the cold is severe and may progress into a more serious condition? |  |  |  |  |  |
| **Information Searches** |  |  |  |  |  |
| Do you search for information about treatment of common colds? |  |  |  |  |  |
| Do you refer to personal experience about common colds? |  |  |  |  |  |
| Do you refer to advice from physicians? |  |  |  |  |  |
| Do you refer to other sources about common colds, such as friends and relatives? |  |  |  |  |  |
| **Alternative Evaluation** |  |  |  |  |  |
| When having common colds, do you choose home remedies for colds (such as staying hydrated and having more rest)? |  |  |  |  |  |
| When having common colds, do you choose self-medication without antibiotics (such as such as 999 Cold Remedy Granules?) |  |  |  |  |  |
| When having common colds, do you choose antibiotic self-medication? |  |  |  |  |  |
| When having common colds, do you go to see a doctor directly? |  |  |  |  |  |
| **Antibiotics Obtainment** |  |  |  |  |  |
| Do you obtain antibiotics from physicians’ prescriptions? |  |  |  |  |  |
| Do you obtain antibiotics from retail pharmacies without prescription? |  |  |  |  |  |
| Do you obtain antibiotics from other ways, such as previous storage? |  |  |  |  |  |
| **Antibiotics Consuming** |  |  |  |  |  |
| Have you increased the dosage of antibiotics because of the unsatisfying effects? |  |  |  |  |  |
| Have you decreased the dosage of antibiotics out of concern about negative side effects? |  |  |  |  |  |
| Have you stopped taking antibiotics when you feel that the symptoms turned better? |  |  |  |  |  |
| **Postuse Evaluation** |  |  |  |  |  |
| Antibiotics have a good effect in treating colds. |  |  |  |  |  |
| The side effects of antibiotics are severe. |  |  |  |  |  |
| The effects of antibiotics are better than those of other medicine. |  |  |  |  |  |
| I will use antibiotics for treating common colds in the future. |  |  |  |  |  |

**Table 2. Measures on influencing factors of antibiotic use based on COM-B framework**

| **Capacity** | | | | | | | |
| --- | --- | --- | --- | --- | --- | --- | --- |
| **Knowledge** | **True** | | **Wrong** | | | **I don’t know** | |
| 1. Antibiotics are effective for treating common colds. |  | |  | | |  | |
| 2. Antibiotics are anti-inflammatory drugs. |  | |  | | |  | |
| 3. Antibiotics are effective for treating viral colds. |  | |  | | |  | |
| 4. Antibiotics are effective for treating bacterial colds. |  | |  | | |  | |
| 5. The human body may become resistant to antibiotics. |  | |  | | |  | |
| 6. Bacteria may become resistant to antibiotics. |  | |  | | |  | |
| 7. Overuse of antibiotics may lead to antibiotic resistance. |  | |  | | |  | |
| 8. Antibiotic resistance does not occur as long as the duration of use is short. |  | |  | | |  | |
| **Self-efficiency** | **Strongly**  **Agree** | **Agree** | | **Neither** | **Disagree** | | **Strongly**  **Disagree** |
| 1. I think I know enough about irrational antibiotic use. |  |  | |  |  | |  |
| 2. I consider myself capable of using antibiotics to treat minor symptoms. |  |  | |  |  | |  |
| 3. I usually have confidence in my self-diagnosis and treatment of colds. |  |  | |  |  | |  |
| 4. I usually know when I need to use antibiotics. |  |  | |  |  | |  |
| 5. I usually know whether I need antibiotics to treat common colds before seeing a doctor. |  |  | |  |  | |  |
| **Opportunity** | | | | | | | |
| **Accessibility of Antibiotics** | **Strongly**  **Agree** | **Agree** | | **Neither** | **Disagree** | | **Strongly**  **Disagree** |
| 1. I can easily buy antibiotics from retail pharmacies |  |  | |  |  | |  |
| 2. I have never been asked to show a prescription from a doctor when purchasing antibiotics at a pharmacy. |  |  | |  |  | |  |
| 3. I can easily get antibiotics from family, friends, and home storage. |  |  | |  |  | |  |
| **Social Influence** | **Strongly**  **Agree** | **Agree** | | **Neither** | **Disagree** | | **Strongly**  **Disagree** |
| 1. I have been recommended to use antibiotic to treat common colds by my friends and family. |  |  | |  |  | |  |
| 2. I have been recommended to buy antibiotics to treat common colds by clerks in retail pharmacies. |  |  | |  |  | |  |
| **Motivation** | | | | | | | |
| **Expected Effects of Antibiotics** | **Strongly**  **Agree** | **Agree** | | **Neither** | **Disagree** | | **Strongly**  **Disagree** |
| 1. Antibiotics can relieve symptoms of common colds. |  |  | |  |  | |  |
| 2. Antibiotics accelerate my recovery from colds. |  |  | |  |  | |  |
| 3. Antibiotics can reduce complications of common colds. |  |  | |  |  | |  |
| 4. Antibiotics can prevent colds from getting worse. |  |  | |  |  | |  |
| **Expected side effects of antibiotics** | **Strongly**  **Agree** | **Agree** | | **Neither** | **Disagree** | | **Strongly**  **Disagree** |
| 1. Antibiotics have few side effects. |  |  | |  |  | |  |
| 2. I am worried about the side effects of antibiotics. |  |  | |  |  | |  |
| 3. I am worried that taking antibiotics regularly will reduce its effectiveness. |  |  | |  |  | |  |
| **Choices of Treatment** | **Strongly**  **Agree** | **Agree** | | **Neither** | **Disagree** | | **Strongly**  **Disagree** |
| 1. I take antibiotics for colds just in case, even though I am unsure of their effectiveness. |  |  | |  |  | |  |
| 2. I believe that taking antibiotics will not do any harm to my body, even though I am unsure of their effectiveness. |  |  | |  |  | |  |
| 3. It is better to use antibiotics than not, even though I am unsure of their effectiveness. |  |  | |  |  | |  |
| **Perceived Threat of URTIs** | **Strongly**  **Agree** | **Agree** | | **Neither** | **Disagree** | | **Strongly**  **Disagree** |
| 1. Every time I catch a cold, I get very worried about my health. |  |  | |  |  | |  |
| 2. Every time I catch a cold, I'm afraid it might develop into a more serious illness. |  |  | |  |  | |  |
| 3. I often worry that a cold might make me very sick. |  |  | |  |  | |  |
| **Perceived Threat of AMR** | **Strongly**  **Agree** | **Agree** | | **Neither** | **Disagree** | | **Strongly**  **Disagree** |
| 1. Antibiotic resistance (superbugs) is a serious problem in China. |  |  | |  |  | |  |
| 2. Antibiotic resistance may threaten the health of me and my family members. |  |  | |  |  | |  |
| 3. I am worried that superbugs could harm me and my family. |  |  | |  |  | |  |
| 4. Reducing antibiotic abuse by individuals plays an important role in reducing antibiotic resistance. |  |  | |  |  | |  |
| 5. I can do something to reduce antibiotic abuse. |  |  | |  |  | |  |

**Table 3. Demographic characteristics**

| 1.Gender | □Male □Female |
| --- | --- |
| 2.Age | _________ |
| 3.Education | □Primary school and below □Junior school and equivalent  □Senor school and equivalent □Junior college □Bachelor's degree  □Master degree □Doctoral degree |
| 4.Occupation | □Farmer □Worker □Student □Healthcare worker □Teacher □Government agencies and enterprises □Self-employed □Retired □Unemployed □Others |
| 5. Do you or your family have a medical background? | □Yes □No |
| 6.Annual household income (Chinese Yuan) | □[0, 20,000) □[20,000, 40,000) □[40,000, 60,000)  □[60,000, 80,000) □[80,000, 100,000) □[100,000, 120,000)  □[120,000, 140,000) □[140,000, 160,000) □≥160,000 |
| 7.Medical Insurance | □Urban Employee Basic Medical Insurance  □Medical Assistance for Urban Resident  □New Co-operative Medical Scheme □Others |
| 8.Do you and your family have a chronic disease? | □Yes □No |
| 9.Health status | □Excellent □Good □Moderate □Poor □Very poor |

**Table 4. Results of Scale Reliability**

| **Scales** | **Number of items** | **Cronbach’s alpha** |
| --- | --- | --- |
| Knowledge | 8 | 0.601 |
| Self-efficacy | 5 | 0.780 |
| Antibiotic availability | 3 | 0.655 |
| Social influence | 2 | 0.728 |
| Expected positive effects of antibiotics | 4 | 0.787 |
| Expected negative effects of antibiotics | 3 | 0.658 |
| Rationale of treatment choice | 3 | 0.804 |
| Perceived threat of URTIs | 3 | 0.894 |
| Perceived threat of AMR | 5 | 0.793 |

**Table 5. Model fit index for latent class analysis**

| **Model** | **Log(L)** | **AIC** | **BIC** | **aBIC** | **Entropy** | **LR** | **Minimum class proportion** |
| --- | --- | --- | --- | --- | --- | --- | --- |
| 1 | -9559.7 7 | 19159.53 | 19253.60 | 19190.09 | / | / | / |
| 2 | -9076.22 | 18234.44 | 18427.27 | 18297.07 | 0.771 | 0.001 | 38.53 |
| 3 | -8899.73 | 17923.47 | 18215.07 | 18018.18 | 0.769 | 0.017 | 22.83 |
| **4** | **-8775.29** | **17716.57** | **18106.93** | **17843.36** | **0.756** | **0.003** | **20.62** |
| 5 | -8700.19 | 17608.37 | 18097.50 | 17767.24 | 0.802 | 0.074 | 10.62 |
| 6 | -8648.60 | 17547.20 | 18135.10 | 17738.15 | 0.813 | 0.701 | 10.13 |
| 7 | -8621.33 | 17534.65 | 18221.32 | 17757.68 | 0.835 | 0.335 | 3.27 |

Note. Boldface type indicates the selected model.

AIC= Akaike’s Information Criterion; BIC=Bayesian Information Criterion; aBIC= adjusted Bayesian Information Criterion; LR= Likelihood Ratio.

Lower values of AIC, BIC, and aBIC indicate better model fit. The LR’s significance level of p < 0.05 indicates an enhanced model fit for K classes over K+1 classes. Higher entropy value denotes a more accurate classification of latent classes. An excessively low value for the minimum class proportion suggests an overly detailed distinction among potential subgroups. Combing the above indices, a four-class model was identified in this study.

**Table 6. Classification Accuracy of Four Behavioural Pattern Model**

| **Posterior Probability** | **Results of model classifications** | | | |
| --- | --- | --- | --- | --- |
|  | **Model 1** | **Model 2** | **Model 3** | **Model 4** |
| Model 1 | 0.885 | 0.028 | 0.085 | 0.002 |
| Model 2 | 0.039 | 0.854 | 0.058 | 0.049 |
| Model 3 | 0.065 | 0.051 | 0.837 | 0.046 |
| Model 4 | 0.001 | 0.044 | 0.053 | 0.902 |
